# On the Stinging Organs of the Medusæ

**Published:** 1842-06

**Authors:** 


*On the Stinging Organs of the Medusæ.*—The severe effects produced by these animals are generally noticed in all extended works on poisons. Dr. Wagner, of Gottingen, remarks, that it has not yet been ascertained whether the stinging or burning power of these is to be ascribed to a corrosive liquid or to a mechanical injury. He imagines that his examinations, made at Nice and Villafranca in 1839, will aid in deciding the question.

The origin of the stinging is to be referred to the external surface of the skin. This has a beautiful, brownish, violet and reddish color in various parts; and when separated, which is easily done, there appears the homogeneous, jelly-like substance, which constitutes the real body of the animal. Where the red spots occur, we find, after the skin is detached, round elevations or inequalities like warts.

Between the red grains of pigment are to be observed round balls or bubbles, out of which, frequently, by the aid of a powerful magnifying power, (for this whole organization can only be recognized by the microscope,) fine threads are seen to project. These threads, spirally rolled up, often come out of themselves, but always do so on the application of a slight pressure. The balls or capsules, which contain these threads, are very loosely attached and easily fall, and are rubbed off along with the slime when the medusa loses its skin; they are found in quantity, as are also the threads themselves in what is termed the stinging slime of the medusa, as is easily ascertained when these animals are kept in vessels.

The slightest touch of a medusa causes a perceptible burning sensation, and this is more or less severe, according to the vigor of the animals. They only sting from parts of their bodies where the epidermis is preserved. Dr. Wagner never experienced the sensation when he came in contact with portions in which the epidermis had been removed. In his case, a burning sensation was felt in from a few seconds to a minute after contact; after a few minutes, a slight redness appeared in his case, and then a simple, lentil-shaped elevation; but, more frequently, three or four near one another. The appearance sometimes resembles nettle-rash. The pain generally soon ceases, but it lasted half a day with one of the party, Dr. Will, and, after eight days, a redness was still perceptible.

The internal substance of the body (jelly) never stings, nor does any part, where the pigment spots, the capsules, and the hairs are wanting.

Dr. Wagner thinks it probable that the stinging has a mechanical and chemical origin, and that the parts above described are poison-organs.—*Amer. Jour. Med. Sci.*, from *Edin. New Philosophical Jour.*, Jan. 1842.

